# Effect of continuous intraoperative infusion of methoxamine on renal function in elderly patients undergoing gastrointestinal tumor surgery: a randomized controlled trial

**DOI:** 10.1186/s12871-020-01064-0

**Published:** 2020-06-13

**Authors:** Xiaowei Guo, Jie Hu, Hanbing Xiao, Tianyu Liu, Zheng Niu, Min Wang, Dunyi Qi

**Affiliations:** 1 Department of Anesthesiology, Affiliated Hospital of Xuzhou Medical University, Key Laboratory of Anesthesia and Analgesia, Xuzhou Medical University, Xuzhou, Jangsu China; 2 Xuzhou Medical University and Department of Oncology, Affiliated Hospital of Xuzhou Medical University, Xuzhou, Jangsu China

**Keywords:** Methoxamine, Elderly patients, Renal function, Gastrointestinal tumor surgery

## Abstract

**Background:**

Acute renal injury (AKI) caused by hypotension often occurs in elderly patients after gastrointestinal tumor surgery. Although vasoactive drugs can increase effective filtration pressure, they may increase renal vascular resistance and reduce renal blood flow. The effect of methoxamine on renal function is not clear.

**Methods:**

After obtaining written informed consent, 180 elderly patients undergoing elective gastrointestinal tumor surgery were randomly allocated into two groups: M group (continuous infusion of methoxamine at 2 μg/kg/min) and N group (continuous infusion of normal saline). The patients’ mean arterial pressure was maintained within 20% of baseline by a continuous infusion of methoxamine or normal saline. Maintenance fluid was kept at 5 mL/kg/h. According to Kidney disease improve global outcome (KDIGO) guidelines, creatinine was measured at 1, 2 and 7 days after operation, and urine volume at 6, 12 and 24 h after operation was measured to evaluate the occurrence of AKI. 162 patients were included in the final data analysis.

**Results:**

Significant differences in the incidence of postoperative Acute kidney injury (M group: 7.5%; N group: 18.3%; *P* < 0.05), the frequency of hypotension (M group: 1 [1–3]; N group: 3 [1–5]; *P* < 0.05), and the duration of intraoperative hypotension (M group: 2[0–10]; N group: 10 [5–16]; *P* < 0.05) were identified between the groups. Multivariate logistic regression analyses demonstrated that preoperative creatinine and the frequency of intraoperative hypotension were the common factors leading to the occurrence of postoperative AKI. The results of Cox multivariate analysis showed that age and AKI were independent risk factors for 30-day death.

**Conclusion:**

Compared with the intraoperative continuous infusion of placebo and methoxamine, continuous infusion of 2 μg/kg/min methoxamine reduced the incidence of postoperative AKI and other clinical complications in elderly patients undergoing gastrointestinal surgery by raising blood pressure and improved the prognosis of patients.

**Trial registration:**

Trial registration: Chinese Clinical Trial Registry, ChiCTR1900020536, registered 7 January, 2019,

## Background

Gastrointestinal malignant tumor are the main cause of morbidity and mortality worldwide, and the elderly have a high incidence. Elderly patients undergoing gastrointestinal tumors surgery are prone to a variety of postoperative complications (such as pneumonia, wound infection, deep venous thrombosis, renal function injury, etc.). Acute renal injury (AKI) is a serious postoperative complication that prolongs the hospitalization time, increases the hospitalization cost and reduces the postoperative survival rate [1–5]. Current studies have shown that the incidence of AKI after gastric and colorectal surgery is 14.4 and 11.8%, respectively [6, 7]. An epidemiological survey of AKI in China showed that the incidence of AKI in patients aged 65 to 80 years old is 15.44%, and the incidence of AKI in patients over 80 years old is 22.22% [8].

Due to poor vascular elasticity and high sensitivity to anesthetics, the elderly are prone to hypotension under anesthesia [9]. Perioperative hypotension has recently been considered an important determinant of postoperative AKI [10, 11]. Blood pressure fluctuations can easily lead to insufficient perfusion of vital organs, although the kidney can maintain self-regulation within a certain range of blood pressure, but the threshold may change with age, so the use of appropriate vasoactive drugs can maintain a certain effective filtration pressure. Methoxamine is a highly selective α_1_ receptor agonist that raises blood pressure and causes the heart rate to slow down, which can increase coronary blood flow, so it is beneficial to improve myocardial hypoxia and is suitable for the elderly. However, previous studies have shown that vasoconstrictors may increase renal vascular resistance and reduce renal blood flow. The effect of methoxamine on renal function is not clear. Therefore, we hypothesized that continuous infusion of methoxamine can maintain renal blood flow and reduce the incidence of postoperative acute renal injury by increasing perfusion pressure in elderly gastrointestinal tumor surgery patients.

The purpose of this study was to investigate the effect of continuous intraoperative infusion of methoxamine on postoperative renal function in elderly patients and whether combined goal-directed fluid therapy can promote the recovery of gastrointestinal function and improve prognosis. The primary outcome was the incidence of postoperative AKI.

## Methods

### Subjects and study design

This study is a single-center, double-blind, prospective, randomized controlled study that has been approved by the Ethics Committee of the Affiliated Hospital of Xuzhou Medical University (the reference number: XYFY2019-KL004, approval date: January 24,2019) and registered in the China Clinical Trials Registry (ChiCTR1900020536). The study was performed from February 2019 to October 2019 at the affiliated Hospital of Xuzhou Medical University. Every participant provided written informed consent before entering the trial. The results are reported in a manner consistent with the CONSORT statement [12].

### Participants

Patients with American Society of Anesthesiologists physical status I–III,aged over 65 years,BMI < 28 kg/cm^2^,who were scheduled for resection of gastrointestinal tumor under general anesthesia were enrolled in this study. The following exclusion criteria were employed: 1) severe hepatorenal insufficiency;2) previous history of hyperthyroidism and pheochromocytoma, severe cardio-cerebrovascular disease,hypertension grade III (very high risk),or hemodynamic instability;3) chronic obstructive pulmonary disease (COPD) who needed bronchodilators and pulmonary hypertension; 4) recent use of tricyclic antidepressants or monoamine oxidase inhibitors or use of nonsteroidal anti-inflammatory drugs in the past month;5) emergency operation.

### Randomization, blinding and allocation concealment

Random sequences were generated by SPSS23.0, and each individual was randomly assigned to the methoxamine group (M group) or placebo group (N group) in a 1:1 ratio. Allocation concealment was conducted by placing a random sequence in opaque, sealed envelopes that were opened after each participant entered the operating room to determine the group assignments. We masked the trial to all participants, investigators, assessors, and statisticians.

### Intervention

All participants fasted for 6 h and forwent drinking for 4 h before surgery. When the participants entered the operation room, each patient was assigned to a specific group after another researcher opened the envelope in a sequential fashion. Researcher prepared the right syringe with a blank label in advance (syringe 1: 1 ml, 10 mg methoxamine + 19 ml 0.9% NaCl; syringe 2: 20 ml 0.9% NaCl) and handed it to the anesthetist involved in the surgery. Methoxamine was infused at 2 μg/kg/min 2 min before induction until the end of operation in the M group. The N group had no prophylactic medication, and normal saline was infused from 2 min before induction to the end of the operation.

After the patient’s arrival at the operating theatre, venous access was opened, ECG and SpO_2_ were monitored. Radial artery catheterization was performed to monitor systemic blood pressure (SBP), diastolic blood pressure (DBP) and mean arterial pressure (MAP). Continuous monitoring of stroke volume variation (SVV) using FloTrac sensor and Vigileo monitor and bispectral index (BIS) was used to monitor the depth of anesthesia. Nasopharyngeal temperature was monitored with a body temperature probe.5 ml/kg/h lactate ringer’s solution was continuously injected during the operation. When SVV > 13%, hydroxyethyl starch was injected within 10 to 15 min until SVV < 13%. If Hb < 80 g/L, we supplemented with blood products. After induction with midazolam 0.03 mg/kg, etomidate 0.3 mg/kg, sufentanil 0.5 μg/kg and cisatracurium 0.15 mg/kg, all patients were intubated with an endotracheal tube and maintained with balanced anesthesia using desflurane 1 MAC, remifentanil (0.05 ~ 0.2 μg/kg/min) and cisatracurium 0.1 mg/kg/h. BIS 40 ~ 60 was maintained by adjusting the drug dosage,insulating blanket was used to keep body temperature at least 36 °C and pneumoperitoneum pressure was maintained between 10 ~ 12 mmHg during the surgery.

MAP was maintained within the range of ±20% of the baseline value. Methoxamine or normal saline infusion was stopped after excluding the cause of shallow anesthesia (BIS> 60) if the patient was in hypertension (> 20% above the baseline value). Methoxamine or normal saline infusion was continued when the blood pressure value was reduced to the baseline value. When the patient was in hypotension (< 80% of the baseline value), ephedrine 6 ~ 10 mg was given to the patients with excessive anesthesia (BIS< 40) and insufficient infusion (SVV > 13%) until the patient reached within 20% of the basal blood pressure level.

### Outcome measurements

The primary outcome was the incidence of postoperative AKI assessed by Kidney Disease: Improving Global Outcomes (KDIGO) criteria [13], AKI was definited as one of the following: when (1) An increase in serum creatinine by ≥0.3 mg/dl (≥26.5 μmol/l) within 48 h.(2) An increase in serum creatinine to ≥1.5 times baseline within the previous 7 days (3) Urine volume ≤ 0.5 ml/kg/h for 6 h. Serum creatinine was measured at 1, 2 and 7 days after surgery, and urine volume was measured at 6, 12 and 24 h after surgery. Secondary outcome were recorded as follows: SBP, DBP, MAP after entering the operation theater (T_0_), 2 min before anesthesia induction (T_1_), 10 min after intubation (T_2_), 30 (T_4_),60 (T_5_), 120 min (T_6_) after beginning the operation, and at the end of operation (T_7_). (2) Intraoperative adverse events: hypotension, hypertension, bradycardia and the use of vasoactive drugs. (3) Intraoperative fluid use, crystal volume, colloid volume, total volume, blood loss and urine volume. 4) The incidence of postoperative complications (post operative nausea and vomiting, incision or abdominal infection, cerebral infarction, pneumonia, myocardial infarction), the time to first exhaust and defecation, postoperative hospital stay and 30-day all-cause mortality.

### Sample size

The calculation of sample size was conducted according to the primary outcome (the incidence of postoperative AKI) with PASS 11.0 (NCSS, LLC, Kaysville, USA). According to previous studies, the incidence of postoperative AKI in elderly surgical patients is 15.44% [8]. We regarded the incidence of AKI in the intervention group decreases to 3% as a clinically meaningful difference. A total of 153 patients were required to achieve 90% power with an α of 0.05 based on the module of analysis of variance (ANOVA) in PASS [14]. Accounting for a 15% dropout rate, each group needed 90 patients in this trial.

### Statistical analysis

For continuous data, Shapiro-Wilk test was used to assess the normality. Normally distributed continuous data were presented as the means (SD), and nonnormal data were presented as medians (interquartile range). Binary data were presented as number (percentage).ANOVA and Mann Whitney-U test were used for normally and nonnormally distributed continuous data. For repeatedly measured outcomes,repeated-measures analysis of variance was used. Binary outcomes were compared using χ2 tests or Fisher exact tests between groups. Rank sum test is used for rank data. Multivariate logistic regression analyses were applied for the primary outcome (AKI). First, multiple collinearity between independent variables was diagnosed according to tolerance or variance inflation factor (VIF). A univariate logistic regression model was used to enter all variables into the model for screening and analysis. Univariate logistic regression identified factors with *P* < 0.10 that were included in the subsequent multivariate logistic regression. The Cox proportional risk model was used to evaluate mortality risk factors 30 days after surgery. A *P*-value less than 0.05 was considered statistically significant. All statistical analyses were conducted using SPSS 23.0 (SPSS, Inc., Chicago, IL, USA).

## Results

### Baseline characteristics

**Fig. 1:**
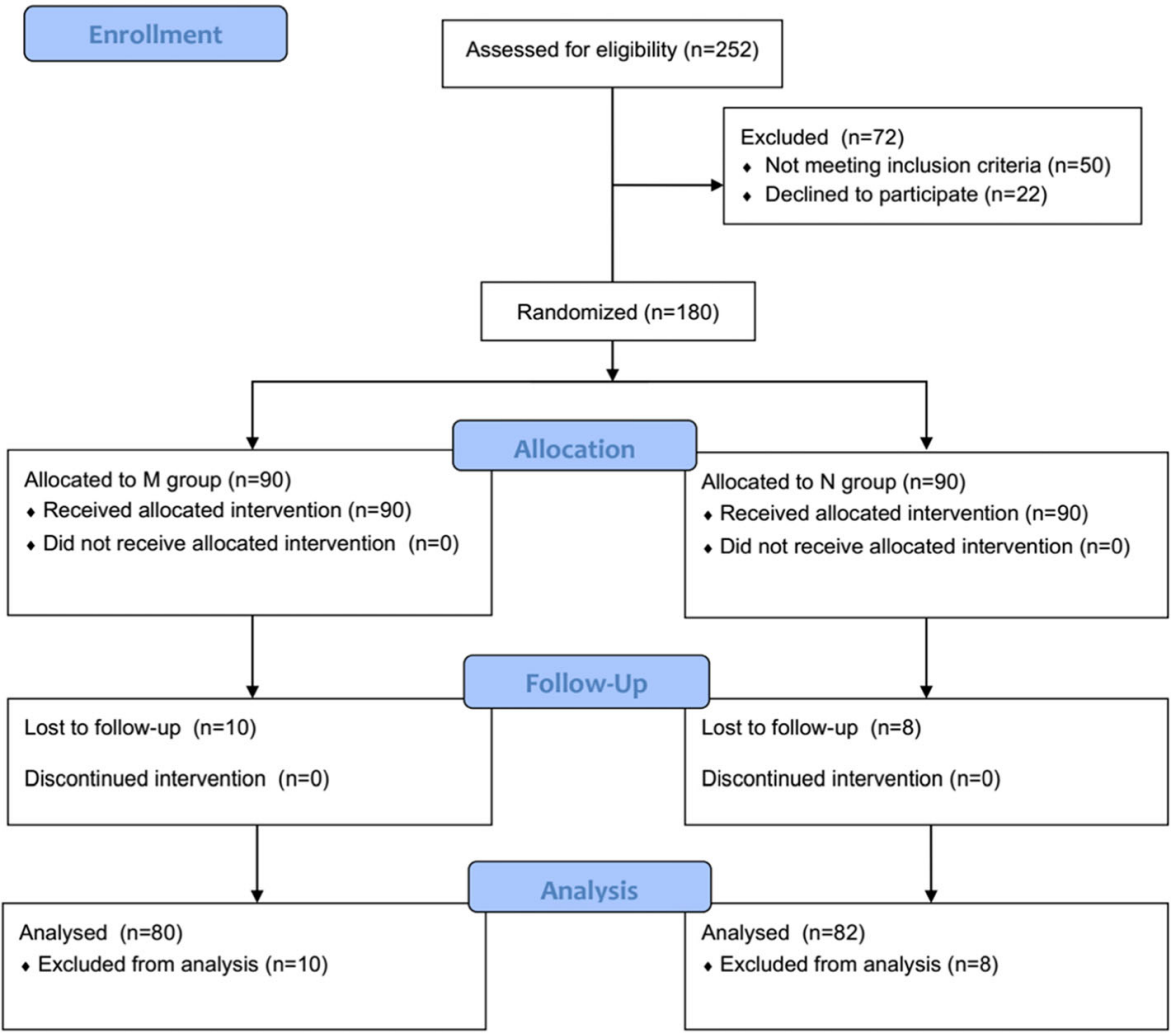
Flow diagram based on Consolidated Standards of Reporting Trials (CONSORT) statement

A total of 252 elderly patients scheduled for elective resection of gastrointestinal tumors were sequentially screened for inclusion between February 2019 and October 2019. A total of 50 participants were excluded according to the inclusion and exclusion criteria, and 22 participants did not give their written informed consent. Finally, 180 patients were randomly allocated to the methoxamine group (M group) and normal saline group (N group) in the proportion of 1:1. Eighteen participants were lost to follow-up in this trial, leaving 80 in M group and 82 in N group in the final analysis. The specific flow diagram of patient selection is presented in Fig. 1. No significant differences in demography, type of operation, disease history, laboratory examination, or baseline hemodynamic characteristics were noted between the two group (Table 1).

**Table 1:** Characteristics of the patients

Variables	M group	N group	*P* value
Age(y)	71.30 ± 5.26	70.61 ± 5.41	0.411
Sex			
Male	50(62.5%)	62(75.6%)	0.071
Female	30(37.5%)	20(24.4%)	
Height (cm)	164.66 ± 7.45	166.66 ± 6.88	0.078
Weight (kg)	63.83 ± 9.84	64.23 ± 9.86	0.796
BMI (kg/cm^2^)	23.50 ± 2.79	23.19 ± 3.03	0.510
Operation type			
Stomach	31(39.2%)	39(48.1%)	0.236
Colon	21(26.6%)	24(29.6%)	
Rectum	27(34.2%)	18(22.2%)	
ASA grade(I/II/III)	14/57/9	19/50/13	0.871
NYHA grade(I/II)	70/10	69/13	0.541
History of smoking	30(37.5%)	34(41.5%)	0.606
History of drinking	26(32.5%)	26(25.5%)	0.299
TIA	12(15%)	15(18.3%)	0.574
COPD	7(8.8%)	12(14.6%)	0.245
Diabetes	7(8.8%)	13(15.9%)	0.169
Hypertension	19(23.8%)	26(31.7%)	0.258
ACEI	3(3.8%)	2(2.4%)	0.680
Hb(g/L)	121.35 ± 21.67	125.52 ± 19.69	0.201
Preoperative creatinine(μmol/L)	64.95 ± 14.67	68.95 ± 16.31	0.096
Preoperative CysC(mg/L)	0.81 ± 0.15	0.94 ± 0.60	0.067
EF(%)	63.40 ± 2.83	62.65 ± 3.43	0.136
Baseline blood pressure			
SBP(mmHg)	124.66 ± 12.99	125.76 ± 12.13	0.576
DBP(mmHg)	74.29 ± 8.67	75.37 ± 7.04	0.386
MAP(mmHg)	90.59 ± 9.23	91.84 ± 8.44	0.368

Data are mean ± SD or number (%)

BMI Body Mass Index, ASA American Society of Anesthesiologists, NYHA New York heart association

TIA Transient Ischemic Attacks, COPD Chronic obstructive pulmonary disease,CysC cystatin C,

SBP Systolic Blood Pressure, DBP Diastolic Blood Pressure, MAP Mean arterial pressure, HR Heart Rate

### Intraoperative hemodynamic outcomes

Compared with the baseline SBP, SBP in the two groups increased significantly at T_0_, T_1_ and T_3_ (*P* < 0.05), and decreased significantly in the N group at T_2_ (*P* < 0.05), SBP in M group was significantly higher than that in group N at T_7_ (*P* < 0.05). Compared with the baseline DBP, except for T_0_, T_1_ and T_3_, the value of DBP in the two groups decreased significantly, and the DBP in M group was significantly higher than that in group N at T_3_-T_7_ (*P* < 0.05). Compared with baseline MAP, MAP in N group decreased significantly except at T_0_, T_1_ and T_3_, MAP in M group decreased significantly at T_2_, and MAP in M group was significantly higher than that in N group at T_2_ and T_4_-T_7_ (*P* < 0.05) (Table 2). The frequency and duration of hypotension in M group were significantly lower than those in N group (*P* < 0.05) (Table 3).

**Table 2:** Intraoperative hemodynamic outcomes

Variables	group	T_0_	T_1_	T_2_	T_3_	T_4_	T_5_	T_6_	T_7_
SBP (mmHg)	M group N group	149.3 ± 20.1^a^ 150.8 ± 21.9^a^	148.4 ± 21.5^a^ 149.5 ± 21.9^a^	119.7 ± 15.5115.8 ± 19.2^a^	138.5 ± 16.9 ^a^ 135.0 ± 20.4^a^	125.0 ± 14.0122.3 ± 15.7	124.0 ± 10.5121.5 ± 16.1	127.5 ± 14.9^b^ 122.1 ± 16.2	137.4 ± 17.9^b^ 125.9 ± 19.1
DBP (mmHg)	M group N group	78.8 ± 10.3 78.3 ± 10.6	75.0 ± 10.9 74.6 ± 11.0	63.2 ± 8.7^a^ 60.0 ± 10.1 ^a^	74.9 ± 11.3^b^ 71.1 ± 12.0 ^a^	69.3 ± 7.7 ^ab^ 65.5 ± 10.8 ^a^	68.8 ± 7.3^ab^ 65.1 ± 8.8 ^a^	69.4 ± 10.1^ab^ 64.4 ± 10.1 ^a^	69.1 ± 11.0 ^ab^ 61.8 ± 11.7 ^a^
MAP (mmHg)	M group N group	102.5 ± 13.2103.2 ± 13.5	100.9 ± 12.0100.3 ± 13.1	82.4 ± 9.5 ^ab^ 78.3 ± 13.1^a^	96.6 ± 13.2 93.1 ± 15.5	88.9 ± 9.6^b^ 83.3 ± 14.6^a^	88.3 ± 7.7^b^ 83.9 ± 11.3^a^	90.4 ± 10.6^b^ 84.0 ± 12.3^a^	94.1 ± 13.3^b^ 83.9 ± 15.1^a^

Data are mean ± SD . ^a^*P* < 0.05 versus baseline; ^b^*P* < 0.05 between the 2 groups

**Table 3:** Intraoperative adverse events

group	hypertension	hypotension	Duration of hypotension	bradycardia
M group	3(1–4)^*^	1(1–3)^*^	2(0–10)^*^	0(0–1)
N group	1(0–2)	3(1–5)	10(5–16)	0(0–1)

Data are median (interquartile range). ^*^P < 0.05 versus N group

### Intraoperative events

There was no significant difference in anesthesia time, operation time, mode of operation, colloid dosage, atropine dosage, urine volume, blood loss, extubation time or departure time between the two groups. The colloid dosage, total fluid volume and ephedrine dosage in group M were significantly less than those in group N (*P* < 0.05) (Table 4).

**Table 4:** Intraoperative events

Variables	M group	N group	*P* value
Anesthesia time (min)	225(190–263)	240(190–280)	0.312
Operation time (min)	195(152–220)	205(154–240)	0.335
Mode of operation (laparotomy/ Laparoscopic)	18/62	20/62	0.777
Liquid dosage (ml)	1678.9 ± 401.3	1899.6 ± 545.5	0.004
Liquid crystal dosage (ml)	1226.9 ± 370.0	1308.5 ± 455.4	0.213
Colloid dosage (ml)	452.1 ± 188.1	591.1 ± 246.4	0.000
Ephedrine dosage (mg)	0(0–6)	6(0–12)	0.001
Atropine dosage (mg)	0(0–0.5)	0(0–1)	0.064
urine volume (ml)	350(208–400)	400(200–500)	0.608
blood loss (ml)	100(100–200)	200(100–262)	0.968
Extubation time (min)	19(10–30)	18(10–30)	0.567
Departure time (min)	45(38–60)	50(40–65)	0.138

Data are mean ± SD or median (interquartile range)

### Postoperative outcomes

The postoperative outcomes of the two groups are presented in Table 5. There was a significant difference in the primary outcome (incidence of AKI) between the two groups (M group: 7.5%;N group: 18.3%, *P* < 0.05). The exhaust time and defecation time in the M group were significantly lower than those in the N group; the incidences of pneumonia in the M group were significantly lower than in the N group. There was no significant difference in the incidence of PONV, incision or abdominal infection, cerebral infarction, ICU admission rate, hospital stay or 30-day mortality between the two groups.

**Table 5:** Postoperative outcomes

Variables	M group	N group	*P* value
evacuation time(d)	2.8(1.8–3.6)	3.4(2.3–4.2)	0.004
defecating time(d)	4.8(3.8–6.2)	6.2(5.2–7.6)	0.000
PONV	7(8.8%)	7(8.5%)	0.961
AKI	6(7.5%)	15(18.3%)	0.041
Incision/abdominal infection	6(7.5%)	5(6.2%)	0.755
Pneumonia	2(2.5%)	10(12.2%)	0.018
cerebral infarction	1(1.2%)	3(3.7%)	0.620
myocardial infarction	0(0%)	2(8%)	0.490
Postoperative admission rate to ICU	3(3.8%)	5(6.1%)	0.491
Postoperative hospital stay(d)	10(8–13)	10.5(9–14)	0.275
30d- mortality	1(1.3%)	5(6.1%)	0.210

Data are median (interquartile range) or number (%)

PONV Post Operative Nausea And Vomitting,AKI acute kidney injury

### Logistic regression for AKI

In this study, these factors were no multicollinearity due to the tolerance was greater than 0.1 and the VIF was less than 10. Univariate regression analysis was run between age, sex, ASA grade, type of operation, past history, preoperative serum creatinine and the incidence of postoperative AKI. The results demonstrated that ASA grade, smoking history, hypertension history, preoperative serum creatinine and the frequency of intraoperative hypotension were the factors leading to postoperative AKI (*P* < 0.1). Then, logistic multivariate regression analysis showed that only preoperative serum creatinine and the frequency of intraoperative hypotension were the common factors leading to postoperative AKI, and the OR values were 1.04 and 1.28, respectively (Table 6).

**Table 6:** logistic regression for AKI

	Univariate	Multivariate		
Variables	OR(95%CI)	*P* value	OR(95%CI)	*P* value
Age	1.00(0.92–1.09)	0.997	–	–
Sex	4.75(1.06–21.26)	0.042	1.98(0.36–10.80)	0.430
ASA grade				
II	5.28(0.67–1.68)	0.114	3.49(0.39–31.44)	0.265
III	8.86(0.99–9.00)	0.051	3.05(0.26–35.62)	0.374
NYHA grade				
II	1.51(0.46–4.97)	0.497	–	–
Operation type				
colon	0.69(0.22–2.15)	0.525	–	–
rectum	0.85(0.29–2.49)	0.772	–	–
COPD	1.54(0.40–5.92)	0.528	–	–
diabetes	1.72(0.52–5.73)	0.375	–	–
TIA	1.13(0.35–3.65)	0.838	–	–
hypertension	2.50(0.99–6.23)	0.052	1.88(0.62–5.66)	0.264
Preoperative creatinine	1.04(1.00–1.07)	0.032	1.04(1.01–1.57)	0.028
Anesthesia time (min)	1.01(0.99–1.01)	0.213	–	–
Operation time (min)	1.01(0.99–1.01)	0.131	–	–
Mode of operation (laparotomy/ Laparoscopic)	0.77(0.29–2.04)	0.598	0.66(0.22–1.93)	0.444
Introperative Liquid volume (ml)	1.00(1.00–1.01)	0.068	1.00(0.99–1.00)	0.446
Introperative urine volume (ml)	1.00(0.99–1.00)	0.990	–	–
Introperative Hb(g/L)	1.14(0.87–1.48)	0.344	–	–
Hypotension frequency	1.30(1.07–1.57)	0.009	1.28(1.04–1.57)	0.020
Duration of hypotension	1.03(0.99–1.07)	0.145	–	–

Factors of *P* < 0.10 in the univariate analysis were included in the multivariate analysis. All the variables for regression analysis were listed in the chart, among which preoperative creatinine and the frequency of intraoperative hypotension were significant risk factors for AKI in this study

OR Odds ratio

### Cox proportional hazards regression analysis

Cox univariate regression analysis was run between age, frequency of hypotension, duration of hypotension, and AKI and 30-day mortality. The results showed that age, frequency of hypotension, duration of hypotension and AKI were correlated with 30-day mortality (*P* < 0.05). The results of cox multivariate analysis showed that age (HR = 1.25, 95% CI: 1.04–1.50) and AKI (HR = 18.12, 95% CI: 2.60–126.39) were independent risk factors for 30-day death (Table 7).

**Table 7:** Cox proportional hazards regression analysis(30-d mortality)

Prognostic Factor	Univariate analysis		*P* value	Multivariate analysis		*P* value
	Unadjusted HR	95% confidence interval		HR	95% confidence interval	
Age	1.24	1.09–1.42	0.001	1.25	1.04–1.50	0.017
Hypotension frequency	1.55	1.22–1.96	0.000	1.19	0.85–1.65	0.311
Duration of hypotension	1.07	1.02–1.12	0.004	1.03	0.95–1.11	0.513
AKI	14.57	2.66–79.56	0.002	18.12	2.60–126.39	0.003

Factors of *P* < 0.05 in the univariate analysis were included in the multivariate analysis. All the variables for regression analysis were listed in the chart, among which Age and AKI were significant risk factors for 30-d mortality in this study

HR Hazard ratio

## Discussion

Our results demonstrated that continuous intraoperative infusion of 2 μg/kg/min methoxamine raised blood pressure and reduced the incidence of postoperative AKI and other clinical complications in elderly patients undergoing gastrointestinal tumor surgery compared with the normal saline group and improved the prognosis of elderly patients.

Recently, perioperative hypotension has been considered to be an important determinant of postoperative AKI. In this RCT, the incidence of AKI in the M group was significantly lower than that in the N group (M group: 7.5%; N group: 18.3%; *P* < 0.05). We observed the level of MAP in group M was controlled within the range of 80 ~ 95 mmHg at each time point. At T_2_ and T_4_-T_7_, the MAP in M group was significantly higher than that in N group (*P* < 0.05), and the frequency and duration of hypotension in N group were significantly higher than those in M group. A single-center cohort study showed that postoperative stage I AKI was associated with intraoperative MAP less than 60 mmHg for more than 20 min and less than 55 mmHg for more than 10 min [10]. A prospective randomized controlled trial showed that in elderly patients, a higher target MAP of 80 to 85 mmHg could reduce AKI after major abdominal surgery [15]. In this study, Logistic regression analysis of AKI also showed that the frequency of intraoperative hypotension was a risk factor for postoperative AKI (OR = 1.28, 95% CI: 1.04–1.57). Hypotension can easily lead to renal ischemic and hypoxic injury mainly related to the following physiological mechanisms: The medulla of the kidney is in a state of low oxygen supply and high oxygen uptake, and the blood supply pressure of the inner medulla part is significantly lower than that of the cortical part. When anemia and hypotension occur, the renal medulla is prone to hypoxia. With increasing age, there will be varying degrees of vascular sclerosis, and the blood pressure-blood flow setting point of important organs will move up so that the kidneys that maintain the level of hypertension may attain better blood perfusion.

Previous studies have shown that renal arteries have a relatively high density of α_1_ receptors [16], and methoxamine may cause renal vessels to constrict to some extent. An animal experiment showed that when the dose of methoxamine was increased from 5 μg/kg/min to 50 μg/kg/min in healthy conscious dogs, renal blood flow decreased by 13 to 37% [17]. However, previous studies used a too-high dose of methoxamine, and no related clinical studies have reported significant renal function damage. Current studies have shown that when vasoconstrictors increase renal perfusion pressure, renal vascular resistance increases to a greater extent through pressure-dependent self-regulation and only partly through α-receptor-mediated vasoconstriction [18]. Despite the changes in MAP, the kidney controls the tension of afferent arterioles through its own regulatory mechanism, keeping renal blood flow (RBF) and glomerular Filtration Rate (GFR) almost unchanged [19, 20]. Previous studies of other α-receptor agonists have also found positive effects on renal function [21–24], the mechanism of renal protection was that it suppressed the excitation of renal sympathetic nerve by increasing the reflex of MAP, increased the expression of cyclooxygenase-2 isoforms in the kidney, weakened the renal vasoconstriction induced by α-receptor agonists, and increased renal blood perfusion.

Several previous large studies have shown that risk factors for postoperative AKI may be associated with the following: age, male sex, BMI, hypertension, preoperative renal insufficiency, higher ASA grade, blood transfusion, preoperative dehydration, colectomy and use of nephrotoxic drugs [25–30]. Consistent with the results of previous studies, this study included a high-risk elderly population with a history of hypertension, and the incidence of postoperative AKI was 18.3%, higher than that of other noncardiac elective surgeries (11.8%) [31]. The logistic multivariate analysis of AKI showed that preoperative high creatinine value and the frequency of intraoperative hypotension were risk factors for AKI, corresponding with the results of previous studies. In this RCT, the amount of operative blood loss was less, and the intraoperative Hb was maintained at a high level. Only two patients received blood transfusion during the operation, and no AKI occurred. The use of intraoperative antibiotics and postoperative analgesics does not require aminoglycoside antibiotics and nonsteroidal anti-inflammatory drugs, and there was no significant difference in the use of ACEI between the two groups. In this study, logistic regression showed that colloids were not a risk factor for AKI. A meta analysis suggested that resuscitation with hydroxyethyl starch could increase the incidence of AKI and the risk of renal replacement therapy in patients with sepsis [32], but this study was for patients undergoing elective major abdominal surgery rather than sepsis. Recently, several studies have shown that the intraoperative use of moderate doses of 6% HES 130/0.4 was not associated with increased risk of AKI [33].

GDFT combined with α_1_ adrenergic receptor agonist can maintain vital organ perfusion without excessive fluid [34]. In this study, by reducing infusion, the time of postoperative exhaust and defecation was significantly shortened, and the incidence of pneumonia was reduced. In this study, there was no myocardial infarction in the M group, which may be because DBP in the M group was significantly higher than that in the N group during T_3_-T_7_, coronary blood flow was effectively increased by increasing DBP, and coronary vessels mainly express α _1D_ receptors, while methoxamine did not act on α_1D_ receptors, so it did not cause coronary vasoconstriction and played a role in myocardial protection [35].

Our study showed that AKI was an independent risk factor for death at 30 days after operation (*P* = 0.003), and the risk of death was 18.12 times higher than that of non-AKI patients (95% CI: 2.60–126.39). A previous prospective observational study showed that patients undergoing noncardiac surgery were 8.3 times more likely to die within 30 days after AKI than other patients (95% CI: 6.2–11.2), [36]. In addition to AKI, age was an independent risk factor for 30-day mortality. This study also showed that although hypotension was associated with the occurrence of AKI, it was not associated with 30-day mortality.

There are limitations to our research. First, the participants in our RCT were elderly patients and were mostly male, and there may be urinary tract obstruction caused by benign prostatic hyperplasia after surgery. We did not conduct urinary color ultrasound screening, while the diagnostic criteria of AKI are based on urine volume and creatinine, which may lead to diagnostic errors. Second, In this study, postoperative cerebral infarction was ischemic stroke. Although the difference was not statistically significant, there was no monitoring of cerebral blood flow during the operation, and the effect of methoxamine on cerebral blood flow in patients should be further explored in future studies. Third, We ignored the use of contrast media in patients, which may affect renal function. Finally, although the mode of operation in this study included endoscopy and laparotomy, the data of the two groups were evenly distributed. After univariate and multivariate logistic regression, it was found that the mode of operation had no effect on AKI, and there was no multicollinearity between the mode of operation and other independent variables. Even though heterogeneity existed, applicability was enlarged.

## Conclusion

In summary, our study substantiated that intraoperative continuous infusion of 2 μg/kg/min methoxamine can reduce the incidence of postoperative AKI,and combined with goal-directed fluid therapy can improve the prognosis. Therefore, methoxamine was recommended for preventing hypotension in elderly patients undergoing major abdominal surgery.

## Data Availability

The datasets generated during the current study are not publicly available due the regulation of data management of Xuzhou Medical College Affiliated Hospital, but are available from the corresponding author on reasonable request.

## Abbrevations

ASA: American society of anesthesiology.
AKI: Acute kidney injury.
BIS: Bispectral index.
BMI: Body mass index.
CO: Cardiac output.
DBP: Diastolic blood pressure.
KDIGO: Kidney disease improve global outcome.
MAP: Mean arterial pressure.
PONV: Post operative nausea and vomitting.
SBP: Systolic blood pressure.
GDFT: Goal-directed fluid therapy.
